# *In vitro* leishmanicidal effect of Yangambin and Epi-yangambin lignans isolated from *Ocotea fasciculata* (Nees) Mez

**DOI:** 10.3389/fcimb.2022.1045732

**Published:** 2023-01-10

**Authors:** Jéssica Rebouças-Silva, Gabriel Farias Santos, José Maria Barbosa Filho, Andresa A. Berretta, Franciane Marquele-Oliveira, Valéria M. Borges

**Affiliations:** ^1^ Laboratory of Inflammation and Biomarkers, Gonçalo Moniz Institute, Oswaldo Cruz Foundation, Salvador, Bahia, Brazil; ^2^ Faculty of Medicine of Bahia, Federal University of Bahia (UFBA), Salvador, Bahia, Brazil; ^3^ Post-Graduate Program in Natural and Synthetic Bioactive Products, Federal University of Paraíba, João Pessoa, Paraíba, Brazil; ^4^ Laboratory of Research, Development and Innovation, Apis Flora Industrial e Comercial Ltda, Ribeirão Preto, São Paulo, Brazil; ^5^ Laboratory of Research, Development and Innovation, Eleve Science Research and Development, Ribeirão Preto, São Paulo, Brazil

**Keywords:** yangambin, epi-yangambin, *Leishmania*, Leishmaniasis, natural compounds, treatment, lignan, *Ocotea fasciculata* (Nees) Mez

## Abstract

**Introduction:**

Yangambin and epi-yangambin are the main lignans found in Louro-de-Cheiro [*Ocotea fasciculata* (Nees) Mez], a tree native to the Atlantic forests of northeastern Brazil whose leaves and bark are widely used in folk medicine. The present study investigated the leishmanicidal and immunomodulatory effects of both lignans in *in vitro* models of infection by *Leishmania amazonensis* or *Leishmania braziliensis*, both etiological agents of Cutaneous Leishmaniasis in Brazil.

**Methods:**

Bone marrow-derived mouse macrophages were infected with *L. amazonensis* or *L. braziliensis* and then treated for 48 h at varying concentrations of yangambin or epi-yangambin.

**Results:**

Yangambin and epi-yangambin were found to reduce the intracellular viability of either *Leishmania* species in a concentration-dependent manner, with respective IC_50_ values of: 43.9 ± 5 and 22.6 ± 4.9 µM for *L. amazonensis*, compared to IC_50_ values of 76 ± 17 and 74.4 ± 9.8 µM for *L. braziliensis*. In this context, epi-yangambin proved more selective and effective against *in vitro* infection by *L. amazonensis*. However, both lignans were found to distinctly modulate the production of inflammatory mediators and other cytokines by macrophages infected by either of the *Leishmania* species evaluated. While yangambin increased the production of IL-10 by *L. braziliensis-*infected macrophages, both compounds were observed to lower the production of NO, PGE_2_, IL-6 and TNF-α in both *Leishmania* species.

**Discussion:**

The present results serve to encourage the development of novel studies aimed at screening natural bioactive compounds with the hope of discovering new therapeutic options for the treatment of Cutaneous Leishmaniasis.

## Introduction

Leishmaniasis a widespread group of neglected vector-borne tropical diseases caused by parasites of the genus *Leishmania*, which are transmitted to humans through phlebotomine vectors. Cutaneous Leishmaniasis (CL) is the most common clinical presentation of this disease. According to the WHO, in 2019 more than 87% of new CL cases were reported in 10 countries: Afghanistan, Algeria, Brazil, Colombia, Iran (Islamic Republic of), Iraq, Libya, Pakistan, Syria and Tunisia (WHO, 2021). CL can produce multiple round or oval-shaped skin ulcers with prominent erythematous edges and a granular background; lesions may even compromise regions of the nasopharyngeal mucosa, which can lead to disfigurement (Brasil, 2017). In Brazil, *Leishmania amazonensis* and *Leishmania braziliensis* are the main etiological agents of CL (MINISTÉRIO DA SAÚDE/SVS/SINAM NET, 2021).

Since 1945, the first-choice drugs for leishmaniasis treatment have been based on pentavalent antimonials (Sb^5+^) (Lima et al., 2007). In the event of therapeutic failure or inability to use antimonial therapy, a second-choice drug is employed, such as amphotericin B, pentamidine, paromomycin or miltefosine. However, these treatment options present serious disadvantages, such as high cost, toxic effects and increasing reports of therapeutic failure. Thus, the development of new, effective, safer and more accessible treatments is urgent.

The isomers yangambin and epi-yangambin are lignans found in *Ocotea fasciculata* (Nees) Mez (also known as *Ocotea duckei* Vatti-mo-Gil), a tree native to the Atlantic forests of northeastern Brazil, popularly known as Louro-de-Cheiro or Louro-canela. Yangambin has been described as being an antagonist of platelet activation receptor (Jesus-Morais et al., 2000; Castro-Faria-Neto et al., 1995), having antiallergic (Serra et al., 1997) and analgesic (Almeida et al., 1995) properties, exerting protective action against cardiovascular injury and anaphylactic shock (Ribeiro et al., 1996; Tibirica et al., 1996; Araújo et al., 2014), as well as antitumor activity in colorectal cells (Hausott et al., 2003) and in the KB tumor cell line (Bala et al., 2015). Studies have also reported the leishmanicidal effect of yangambin on promastigotes (Neto et al., 2007; Neto et al., 2011) and in intracellular amastigotes, as well as in an *in vivo* model of CL (Penha, 2010). However, few studies have evaluated the leishmanicidal and immunomodulatory properties of yangambin in an *in vitro* or *in vivo* model of infection. Moreover, to date, there are no known studies evaluating the leishmanicidal and immunomodulatory properties of epi-yangambin.

The present study aimed to shed light on the leishmanicidal and immunomodulatory effects of yangambin and epi-yangambin using *in vitro* infection models of *L. amazonensis* and *L. braziliensis*, the main etiologic agents of CL in Brazil.

## Materials and methods

### Material and reagents

Schneider’s insect medium, IFN-γ, Dimethylsulfoxide (DMSO) and Resazurin sodium salt were obtained from SIGMA-Aldrich (St Louis, MO, USA). A Lactate Dehydrogenase (LDH) Cytotoxicity Detection Kit and Nutridoma™-SP media supplement were obtained from Roche Diagnostics GmbH (Sandhofer Strasse, Mannheim, Germany). Inactivated fetal bovine serum (FBS), RPMI 1640 medium and RPMI 1640 medium without phenol red and penicillin were purchased from GIBCO (Carlsbad, CA, USA). Streptomycin and L-glutamine were obtained from Invitrogen (Carlsbad, CA, USA). A PGE_2_ Elisa kit was purchased from Cayman Chemical Company (Ann Arbor, Michigan, USA) and the MILLIPLEX^®^ kit based on Luminex^®^ xMAP^®^ technology was obtained from Merck (Darmstadt, Germany).

### Yangambin and epi-yangambin isolation & preparation

**Figure 1:**
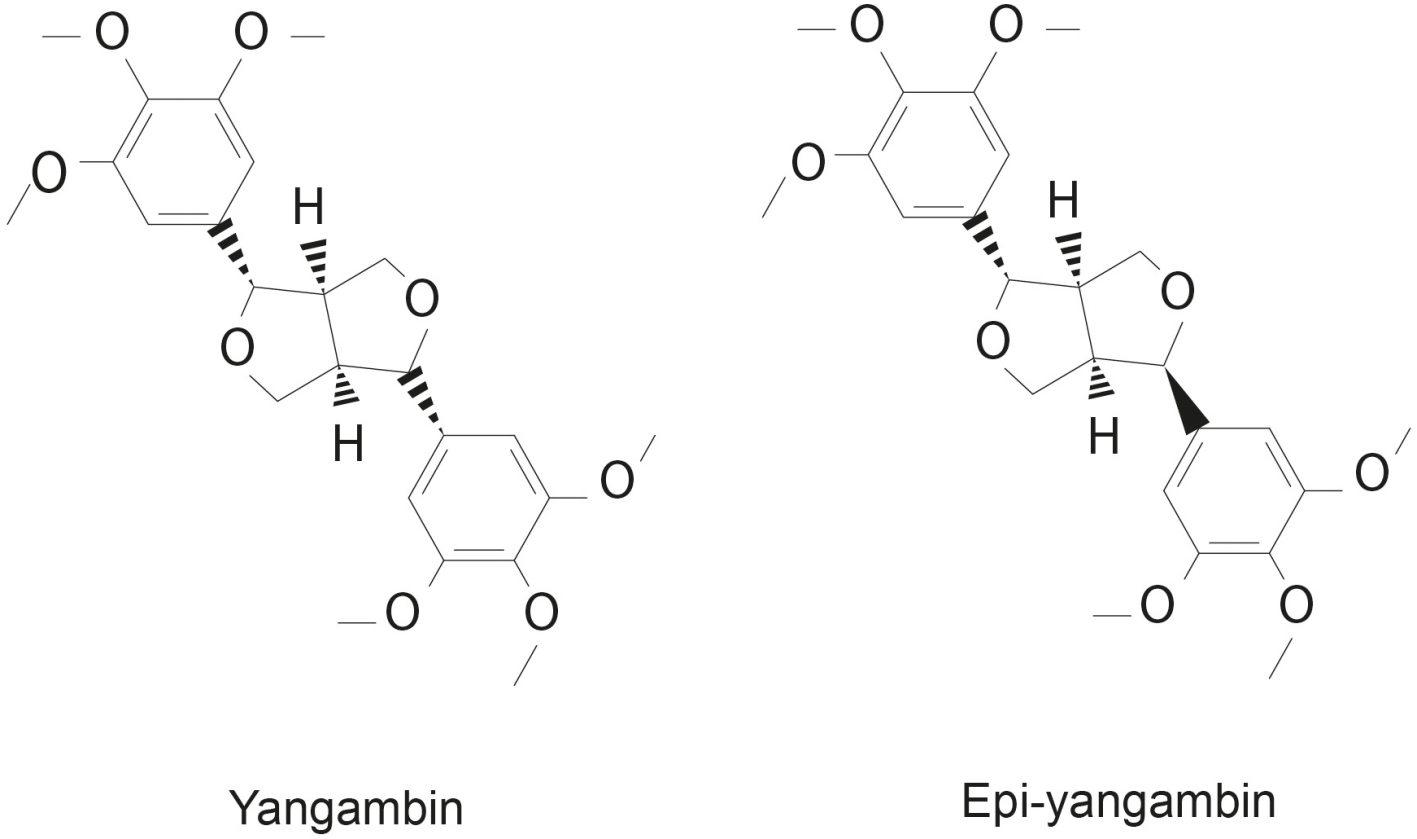
Chemical structures of yangambin and epi-yangambin lignans.

The dried leaves and stem bark of *Ocotea fasciculata* (commonly known as *Louro de Cheiro* in Portuguese) were collected in the state of Paraíba (northeastern Brazil). Both yangambin and epi-yangambin lignans were originally isolated and purified by Martins et al. (2020), who also characterized these compounds using high resolution mass spectrometry (HRMS) and nuclear magnetic resonance (NMR) approaches. The obtained yield was 17% and 29% for yangambin and epi-yangambin, respectively; the chemical formula identified was C_24_H_30_O_8,_ with a corresponding molecular weight of 446 g/mol. Further, a negligible amount of impurities was detected given the quantity of compounds recovered (Martins et al., 2020). A representative illustration of both lignans is shown in **Figure 1**. Subsequently, both lignans were solubilized in DMSO to prepare stock solutions at a concentration of 5 mg/mL.

### Ethics statement

Female BALB/c mice aged 6–8 weeks were obtained from the animal care facility at the Gonçalo Moniz Institute (IGM-FIOCRUZ), located in the city of Salvador, Bahia-Brazil. All animal experimentation was conducted in accordance with the Guidelines for Animal Experimentation established by the Brazilian Council for the Control of Animal Experimentation (CONCEA). The present study received approval from the local institutional review board (CEUA protocol no.: 015/2015, IGM-FIOCRUZ).

### Parasites

Wild-type *L. amazonensis* (MHOM/Br88/Ba-125) and *L. braziliensis* (MHOM/BR/01/BA788) strains were cultured in Schneider’s insect medium supplemented with 10% inactivated FBS, 100 U/mL penicillin, 100 µg/mL streptomycin and 2 mM L-glutamine in 25 cm^2^ flasks at 24°C.

### Macrophage toxicity assay

Bone-marrow derived macrophages (BMDM) were obtained from BALB/c mice femurs and cultured at 37°C under 5% CO_2_ for 7 days in RPMI medium supplemented with 20% FBS, 100 U/mL penicillin, 100 mg/mL streptomycin, 2 mM L-glutamine and 30% L929 cell culture supernatant (a source of macrophage colony-stimulating factor). Next, differentiated BMDM were harvested using cold saline solution. BMDM (1×10^5^/well) were plated on 96-well plates and cultured at 37°C under 5% CO_2_ in RPMI medium without phenol red, supplemented with 1% Nutridoma™-SP (instead of FBS) overnight. BMDM were then treated with either yangambin or epi-yangambin at varying concentrations (15.6–1000 μM) at 37°C for 48h. Uninfected BMDM cultures supplemented with RPMI medium (Medium) or DMSO (~0.1% v/v) (Vehicle) were used as controls. Next, cells were reincubated for another 4 h with supplemented RPMI medium containing 10% Resazurin sodium salt. Absorbance was read at 570 nm and 600 nm using a spectrophotometer (SPECTRAMax 190). Quantification of cytoplasmic lactate dehydrogenase (LDH) enzyme activity was performed in cell-free supernatants following centrifugation at 250×g. Measured using a commercial LDH Cytotoxicity Detection Kit in accordance with the manufacturer’s instructions, LDH absorbance was recorded at 490 nm using a spectrophotometer (SPECTRAMax 190). In addition, Total LDH activity was determined by lysing BMDM with 1% Triton X-100. The percentage of LDH release was calculated as follows: [(LDH sample – Blank LDH) × 100]/Total LDH.

### Assessment of *Leishmania amazonensis* and *Leishmania braziliensis* intracellular viability

BMDM were isolated as described above; 10^5^ cells/well were seeded on 96-well plates in supplemented RPMI medium. Macrophages were then infected (10∶1) with stationary-phase *L. amazonensis* (MHOM/Br88/Ba-125) or *L. braziliensis* (MHOM/BR/01/BA788) promastigotes for 4 h or 24 h, respectively. Next, macrophage cultures were washed 3x with saline to remove any non-internalized parasites and treated with varying concentrations (50 - 200 μM) of yangambin or epi-yangambin for 48 h at 37°C under 5% CO_2_. After replacing the medium with 0.2 mL of supplemented Schneider’s insect medium, cells were cultured at 24°C for an additional 6 days, after which the number of viable parasites was determined by direct counting using a Neubauer chamber.

### Quantification of inflammatory mediators

BMDM (5x10^5^/well) were stimulated with IFN-γ (100 UI/mL) overnight and infected with stationary-phase *L. amazonensis* or *L. braziliensis* promastigotes for 4 h or 24 h, respectively. Macrophages were then washed thrice to remove any non-internalized parasites, the RPMI cell medium was replaced and IFN-γ stimulation was reapplied along with 100 μM of yamgambin or epi-yangambin for 48h. After collecting culture supernatants, the Griess reaction was used to measure nitric oxide (NO) production. Production levels were measured in culture supernatants using a commercially available MILLIPLEX® kit based on Luminex® xMAP® technology (Merck, Darmstadt, Germany) in accordance with the manufacturer’s instructions.

### Statistical analysis

Data are presented as means ± standard deviation (SD) from experiments performed in quintuplicate. Kruskal–Wallis nonparametric testing with Dunn’s post-test was used for multiple comparisons. Comparisons between two groups were performed using the Mann–Whitney non-parametric test. Sigmoidal dose–response curves were used to determine mean inhibition concentration (IC_50_) values relative to intracellular parasite viability, as well as cytotoxicity concentration implying 50% cell viability (CC_50_). The selectivity index (SI), i.e., how selective a compound is to parasites versus macrophages, was determined by calculating CC_50_:IC_50_ ratios. Mean values (Log10) of each mediator were measured in cell supernatants after 48h for each *in vitro* treatment condition. Hierarchical cluster analysis using Ward’s method was performed to determine whether cytokine levels could differentiate between the two treatment conditions (yangambin or epi-yangambin) in the context of either *L. amazonensis* or *L. braziliensis* infection. Heatmaps were built using JMP Pro v.13.0.0. Analyses were performed using GraphPad Prism v.8.00 for Windows (GraphPad Software, San Diego California). Results were considered statistically significant when p <0.05.

## Results

### Yangambin and epi-yangambin lignans exhibit low toxicity

**Figure 2:**
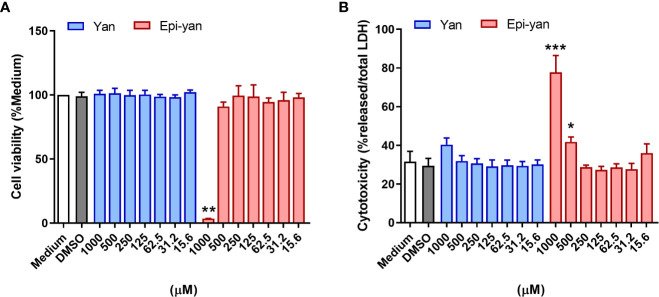
Cytotoxicity of yangambin and epi-yangambin. The cytotoxic potential of both lignans was assessed by **(A)** Sodium resazurin reduction assay and **(B)** total LDH release after 48 h of treatment at varying concentrations (1000 - 15.6 µM). Bars represent ± SD of one representative experiment, from three independent experiments performed in quintuplicate. The Kruskal-Wallis test, followed by Dunn’s post-test, was used for multiple comparisons (*p<0.05, **p<0.01 and ***p<0.0001). Yan, yangambin; Epi-yan, epi-yangambin.

The potential cytotoxic effects of yangambin and epi-yangambin were evaluated in uninfected BMDM *via* sodium resazurin and LDH assays. Under both techniques, only epi-yangambin was able to significantly affect cell viability at the highest concentrations used (1000 and 500 µM), as the highest concentration reduced cell viability by ~97% in sodium resazurin assays (p<0.01) (**Figure 2A**), and induced a ~77% release of LDH (p<0.0001) (**Figure 2B**). Thus, the CC_50_ for epi-yangambin was established at 534 ± 105 µM (**Table 1**). By contrast, treatment with yangambin produced no evident cytotoxic effect in our analysis.

**Table 1:** IC_50_, CC_50_ and Selective Index (SI) values of BMDM infected or not with *Leishmania amazonensis* or *Leishmania braziliensis* and treated for 48 h with yangambin or epi-yangambin.

Compound	*L. amazonensis* (intracellular amastigote) IC_50_ ^a^ ± SEM^b^ (µM)	*L. braziliensis* (intracellular amastigote) IC_50_ ^a^ ± SEM^b^ (µM)	Macrophage CC_50_ ^c^ ± SEM (µM)	SI^d^ *L. amazonensis*	SI^d^ *L. braziliensis*
Yangambin	43.9 ± 5 p=0.057	76 ± 17	ND^e^	ND^e^	ND^e^
Epi-Yangambin	22.6 ± 4.9*	74.4 ± 9.8	534 ± 105	23.6	7.1

a IC_50_: half-inhibitory concentration, i.e., the concentration needed to inhibit a given biological process by half;

b SEM: standard error of the mean;

c CC_50_: 50% cytotoxic concentration, the concentration needed reduce cell viability by 50%;

d SI: selectivity index, as calculated by the ratio: Macrophage CC_50_/amastigote intracellular IC_50_;

e ND: not detected;

*Indicates statistically significant difference (p <0.05) between the IC_50_ of L. amazonensis and L. braziliensis after treatment with epi-yangambin.

### Concentration-response effect of yangambin and epi-yangambin on *Leishmania amazonensis* and *Leishmania braziliensis* intracellular viability

**Figure 3:**
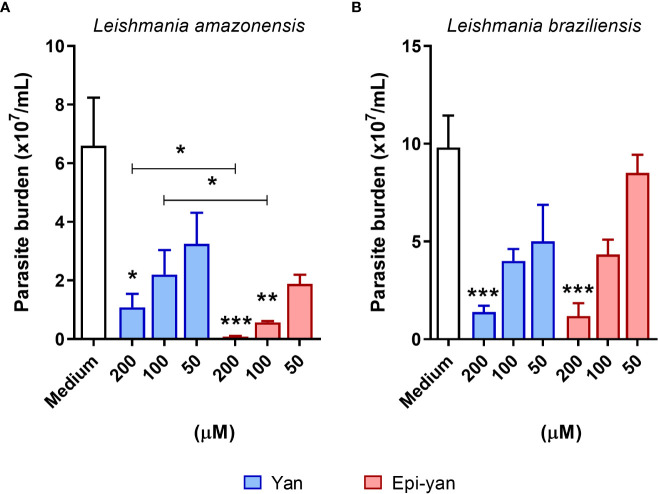
Leishmanicidal effect of yangambin and epi-yangambin on the intracellular viability of *L. amazonensis* and *L. braziliensis*. BMDM were infected with *L. amazonensis* or *L. braziliensis* and treated at varying concentrations of yangambin and epi-yangambin compounds (200 - 50 µM) for 48h. The counting of viable **(A)** *L. amazonensis* and **(B)** *L. braziliensis* promastigotes recovered from culture supernatants was performed six days after changing culture medium, as described in *Materials and Methods*. Bars represent ± SD one representative experiment from four independent experiments performed in quintuplicate. The Kruskal-Wallis test, followed by Dunn’s post-test, was used for multiple comparisons (*p<0.05, **p<0.01 and ***p<0.0001). La, *Leishmania amazonensis*; Lb, *Leishmania braziliensis*; Yan, yangambin; Epi-yan: epi-yangambin.

The leishmanicidal effect of both lignans was evaluated in BMDM infected by *L. amazonensis* or *L. braziliensis* (**Figure 3**). Both lignans were found to reduce the intracellular viability of both *Leishmania* species evaluated in a concentration-dependent manner after 48h of treatment. However, epi-yangambin was shown to be more effective in reducing the intracellular viability of *L. amazonensis* compared to yangambin at similar concentrations (200 and 100 µM) (p< 0.05) (**Figure 3A**). Moreover, epi-yangambin also proved to be more potent against *L. amazonensis* infection compared to *L. braziliensis* infection (p<0.05), as evidenced by the respective IC_50_ values: 22.6 ± 4.9 versus 74.4 ± 9.8 (**Table 1**). Both lignans were found to present moderate and similar leishmanicidal effects against *L. braziliensis* infection (**Figure 3B**). **Table 1** summarizes the CC_50_, IC_50_ and Selectivity Index (SI) values calculated ​for each compound in accordance with either infection condition.

### Immunomodulatory effects of yangambin and epi-yangambin treatment

**Figure 4:**
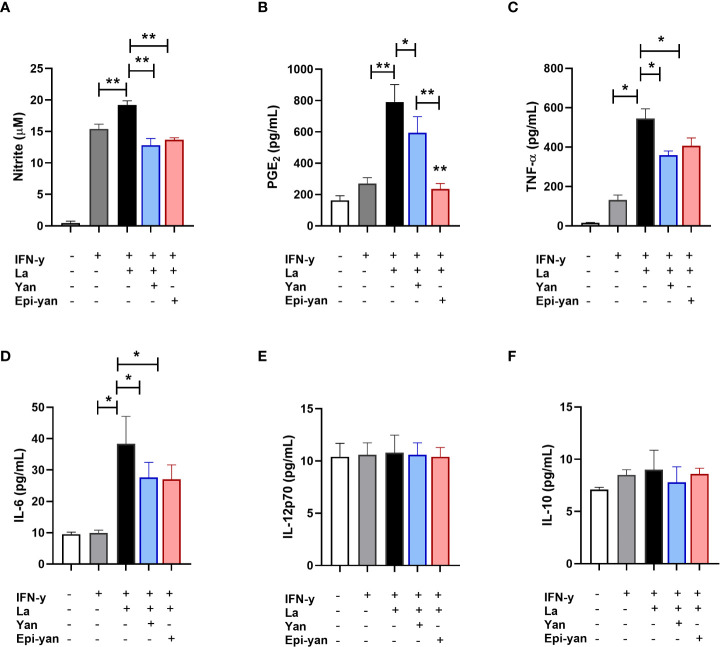
Immunomodulatory effects of yangambin and epi-yangambin on *L. amazonensis*-infected macrophages. Mediators were analyzed in the supernatants of cultured BMDM previously stimulated with IFN-γ, then infected with *L. amazonensis*, followed by treatment with yangambin or epi-yangambin (100 µM) for 48h, as described in *Materials and Methods*. **(A)** Nitrite, **(B)** PGE2, **(C)** TNF-α, **(D)** IL-6, **(E)** IL-12p70 and **(F)** IL-10. Bars represent ±SD of a single experiment performed in quintuplicate. The Kruskal-Wallis test, followed by Dunn's post-test, was used for multiple comparisons, while the Mann-Whitney test was used for comparisons between two groups (*p < 0.05 and **p < 0.01). La, *Leishmania amazonensis*; Yan, yangambin; Epi-yan, epi-yangambin.

The immunomodulatory effects of the yangambin and epi-yangambin lignans were evaluated in the supernatants of macrophages previously stimulated with IFN-y, then infected with *L. amazonensis* or *L. braziliensis*, and finally treated with one of the lignans at 100 µM for 48 h, as described above in the *Materials and Methods* section. In *in vitro L. amazonensis* infection, treatment with either lignan similarly reduced the production of nitric oxide (NO) (p<0.01), TNF-α (p<0.05) and IL-6 (p<0.05) in infected cells, yet no statistical differences were observed in the production of IL-12p70 or IL-10 (**Figure 4**). Although both yangambin and epi-yangambin significantly reduced PGE_2_ levels compared to untreated *L. amazonensis*-infected cells (p<0.05 and p<0.01, respectively), epi-yangambin exerted a more potent effect compared to yangambin (p < 0.01) (**Figure 4B**).

**Figure 5:**
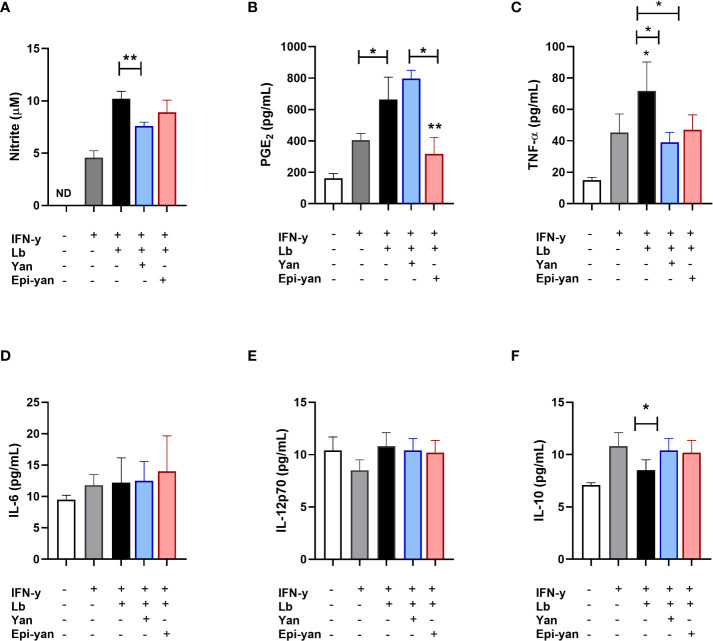
Immunomodulatory effects of yangambin and epi-yangambin on *L. braziliensis*-infected macrophages. Mediators were analyzed in the supernatants of cultured BMDM previously stimulated with IFN-γ, then infected with *L. braziliensis* and finally treated with yangambin or epi-yangambi (100 µM) for 48h, as described in *Materials and Methods*. **(A)** Nitrite, **(B)** PGE2, **(C)** TNF-α, **(D)** IL-6, **(E)** IL-12p70 and **(F)** IL-10. Bars represent ±SD of a single experiment performed in quintuplicate. The Kruskal-Wallis test, followed by Dunn's post-test, was used for multiple comparisons, while the Mann-Whitney test was used for comparisons between two groups (*p < 0.05 and **p < 0.01). Lb, *Leishmania braziliensis*; Yan, yangambin; Epi-yan, epi-yangambin.

Regarding *in vitro L. braziliensis* infection, both lignans were observed to significantly reduce TNF-α levels (p< 0.05). However, only yangambin significantly reduced NO levels (p<0.01) and also increased IL-10 production (p<0.05) in infected macrophages. Again, epi-yangambin was shown to be more potent in reducing PGE_2_ levels, both in relation to the infected control (p<0.01) and to yangambin (p<0.05) (**Figure 5B**). Neither of the lignans were shown to modulate the production of IL-6 or IL-12p70 (**Figure 5**).

**Figure 6:**
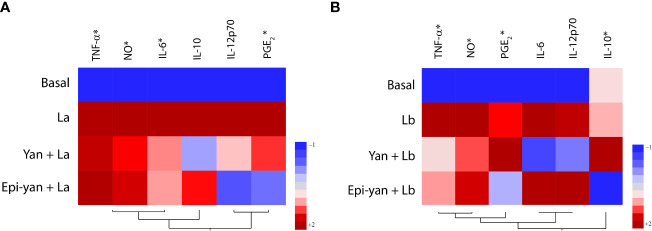
Inflammatory profile of yangambin- and epi-yangambin-treated macrophages infected with either *L. amazonensis* or *L. braziliensis*. Hierarchical clustering analysis (Ward’s method) was used to assess whether cytokine production could differentiate between **(A)** *Leishmania amazonensis* or **(B)** *Leishmania braziliensis*, infection, in cells treated or not with yangambin or epi-yangambin, as described in Materials and Methods. La, *Leishmania amazonensis*; Lb, *Leishmania braziliensis*; Yan, yangambin; Epi-yan, epi-yangambin.

A heatmap was generated to illustrate clustering in the modulation of inflammatory mediators produced by *L. amazonensis* or *L. braziliensis-*infected cells treated or not with yangambin or epi-yangambin (**Figures 6A, B**). This analysis revealed that treatment with either lignan attenuated the inflammatory profile of infected cells, more so in the context of *L. amazonensis* infection.

## Discussion

The thalidomide tragedy in the 1950-60s shed light on the need for greater drug regulation. Introduced in the 1950s as a sedative, tranquilizer and antiemetic for morning sickness, thalidomide was a racemic mixture of two straight (R) and sinister (S) enantiomers, in which the R isomer acts as a sedative, while the S isomer serves as a teratogen; this in turn led to thousands of children being born with malformations in the limbs, ears, heart and internal organs (Moro and Invernizzi, 2017; Kesserwani, 2021). Thus, considering the importance of studying isomers to develop safe and highly effective drugs, the present work builds on previous reports by evaluating the leishmanicidal and immunomodulatory effects of the yangambin and epi-yangambin isomers.

In the present work, our results show that both lignans proved effective in controlling *in vitro* infection by both *Leishmania* species evaluated. Regarding cytotoxicity, epi-yangambin exhibited greater cytotoxicity than yangambin, which is consistent with data reported by Martins et al. (2020). Monte Neto et al. (2008), who evaluated the cytotoxicity of yangambin (24h incubation period) on murine macrophages using Trypan blue exclusion assays and an MTT colorimetric assay, described CC_50_ values ​​of 187 μg/mL (383.8 μM) and 46.7 μg/mL (504.3 μM), respectively. In contrast, we observed no cytotoxic effects following *in vitro* treatment with yangambin in BALB/c macrophages. The discrepancies between the obtained results may be related to the cytotoxicity assay techniques used, specificities in cell types and the degree of purity of the compounds evaluated.

The leishmanicidal effects of the presently investigated lignans remains poorly studied in the scientific literature. Most publications have exclusively reported on the effect of yangambin on promastigote forms of *Leishmania* (Neto et al., 2007; Neto et al., 2011). Although efficacy data on promastigotes does shed light on this lignan’s potential leishmanicidal effect, only by evaluating intracellular amastigote forms can this be confirmed. Herein, our results suggest that epi-yangambin was more potent in reducing the intracellular viability of *L. amazonensis* than yangambin, with respective IC_50_ values ​​of 22.6 ± 4.9 µM and 43.9 ± 5 µM, respectively. Furthermore, epi-yangambin also demonstrated greater specificity for *L. amazonensis* compared to *L. braziliensis* infection, with respective IC_50_ values ​​of 22.6 ± 4.9 µM and 74.4 ± 9.8 µM, respectively, and corresponding SI values of 23.6 and 7.1. According to Batista et al. (2009), compounds presenting IC_50_ values between 20-100 μM are considered to exert moderate activity. In addition, compounds with SI values >10 are considered bioactive, thus deserving further investigation (Indrayanto et al., 2021). Our data indicate that epi-yangambin is more selective and effective at reducing *L. amazonensis* intracellular viability in comparison to *L. braziliensis*. However, yangambin was shown to be less cytotoxic to host cells, which suggests greater selectivity of this compound in killing the intracellular forms of both *Leishmania* species evaluated. To the best of our knowledge, these results are novel in that they demonstrate the leishmanicidal effects of epi-yangambin on intracellular *Leishmania* parasite viability. Furthermore, this study also represents the first attempt to evaluate yangambin in the context of intracellular viability of *Leishmania* species related to CL.

The immunological spectrums of human infection by *L. amazonensis* or *L. braziliensis* species are distinct. *L. amazonensis* infection tends to induce clinical forms associated with the anergic pole of the host’s cellular immune response (Diffuse Leishmaniasis), while *L. braziliensis* alternatively tends to induce cellular hypersensitivity (Mucocutaneous Leishmaniasis). In the middle of this immunological spectrum lies Localized Cutaneous Leishmaniasis (LCL) (Silveira et al., 2004; Silveira et al., 2009). Although the Th1 response (i.e., the production of pro-inflammatory mediators, such as IFN-*y*, TNF-α, ROS, NO, IL-6 and IL-12) contributes to the control of Leishmania infection, an exacerbated Th1 response can lead to severe tissue damage in CL. Thus, a pro-inflammatory response alone is insufficient for the control of infection, since patients with Mucocutaneous Leishmaniasis exhibit an exacerbated inflammatory response with reduced levels of the regulatory cytokine IL-10, yet they are unable achieve cure (Rodrigues et al., 2014).

In this context, drugs and therapeutic regimens exerting parasite control activity, while also balancing pro- and anti-inflammatory responses, are desirable to control this condition. Accordingly, lignans yangambin and epi-yangambin have emerged as promising candidates for future evaluation in the development of new pharmaceutical options for CL treatment. Our data suggest that the leishmanicidal effects produced by both lignans are not related to macrophage activation. Despite the absence of a cellular activation pathway linked to the leishmanicidal effect presented by yangambin and epi-yangambin, our findings support the hypothesis that these compounds may exert a direct effect on intracellular parasite, which deserves further investigation.

Importantly, our results show that both lignans were found to reduce the levels of important pro-inflammatory mediators, e.g., NO, IL-6 and TNF-α, in cells infected by *L. amazonensis* or *L. braziliensis*, while also reducing intracellular viability. These data corroborate a report by Penha (2010) describing the ability of yangambin to reduce NO and TNF-alpha levels in *L. chagasi*-infected cells, a *Leishmania* species related to visceral leishmaniasis (Penha, 2010).

Although a range of pharmacological properties have already been attributed to yangambin and, to a lesser extent, epi-yangambin, the present study offers relevant insight into the immunomodulatory effects of both lignans in *in vitro* infection by *L. amazonensis* and *L. braziliensis*.

## Conclusion

Lignans yangambin and epi-yangambin represent potential candidates for further evaluation in the development of novel treatments against CL. Both lignans demonstrated leishmanicidal effects *in vitro*, accompanied by an attenuated inflammatory response, in macrophages infected by *L. amazonensis* or *L. braziliensis*, both etiological agents of CL in Brazil. In addition, the study of yangambin and epi-yangambin lignans serves to highlight Brazilian biodiversity in the screening of bioactive compounds and molecules for therapeutic applications. It is important to study these compounds, not only for the possibility of discovering new, more affordable treatments, but also to encourage further investigation into the possible mechanisms of action involved in the biological effects identified herein, which open up new possibilities for the development of target-specific drugs.

## Data availability statement

The original contributions presented in the study are included in the article/Supplementary Material. Further inquiries can be directed to the corresponding author.

## Ethics statement

The animal study was reviewed and approved by Brazilian Council for the Control of Animal Experimentation (CONCEA), IGM-FIOCRUZ.

## Author contributions

All authors made substantial contributions to the study’s conception and design, acquisition of data, or analysis and interpretation of data; took part in drafting the article or revising it critically for important intellectual content; gave final approval of the version to be published; and agree to be accountable for all aspects of the work.

## References

[B1] Almeida R. N. Pachu C. Barbosa-Filho J. (1995). Avaliação da possível atividade analgésica da iangambina obtida de ocotea duckei vatimo. Cienc Cult Saúde 14, 7–10.

[B2] Araújo I. G. A. Silva D. F. do Carmo de Alustau M. Dias K. L. G. Cavalcante K. V. M. Veras R. C.. (2014). Calcium influx inhibition is involved in the hypotensive and vasorelaxant effects induced by yangambin. Molecules 19, 6863–6876. doi: 10.3390/molecules19056863 24858272 PMC6271947

[B3] Bala M. Pratap K. Verma P. K. Singh B. Padwad Y. (2015). Validation of ethnomedicinal potential of tinospora cordifolia for anticancer and immunomodulatory activities and quantification of bioactive molecules by HPTLC. J. Ethnopharmacol 175, 131–137. doi: 10.1016/j.jep.2015.08.001 26253577

[B4] Batista R. de Jesus Silva Júnior A. de Oliveira A. B. (2009). Plant-derived antimalarial agents: New leads and efficient phytomedicines. part II. non-alkaloidal natural products. Molecules 14, 3037–3072. doi: 10.3390/molecules14083037 19701144 PMC6254980

[B5] Brasil Ministério da Saúde (2017). *Manual De Vigilância Da Leishmaniose Tegumentar*. Brasília: Ministério da Saúde, Secretaria de Vigilância em Saúde, Departamento de Vigilância das Doenças Transmissíveis.

[B6] Castro-Faria-Neto H. C. Araujo C. Moreira S. Bozza P. T. Thomas G. Barbosa-Filho J. M.. (1995). Yangambin: A new naturally-occurring platelet-activating factor receptor antagonist: *In vivo* pharmacological studies. Planta Med. 61, 106–112. doi: 10.1055/s-2006-958026 7753914

[B7] Hausott B. Greger H. Marian B. (2003). Naturally occurring lignans efficiently induce apoptosis in colorectal tumor cells. J. Cancer Res. Clin. Oncol. 129, 569–576. doi: 10.1007/s00432-003-0461-7 12898234 PMC12161906

[B8] Indrayanto G. Putra G. S. Suhud F. (2021). Validation of in-vitro bioassay methods: Application in herbal drug research. Profiles Drug Subst. Excipients Relat. Methodol. 46, 273–307. doi: 10.1016/bs.podrm.2020.07.005 33461699

[B9] Jesus-Morais M. Assis F. Cordeiro S. Barbosa-Filho M. Lima T. Silva L.. (2000). Yangambin, a lignan obtained fromOcoteaduckei, DifferentiatesPutative PAF receptor subtypes in the gastrointestina lTract of rats. Planta Med. 66, 211–216. doi: 10.1055/s-2000-8556. 10821044

[B10] Kesserwani H. (2021). Death and rebirth of the thalidomide molecule: A case of thalidomide-induced sensory neuropathy. Cureus 13, 13140. doi: 10.7759/cureus.13140 PMC7936918 33728154

[B11] Lima E. B. Porto C. da Motta J. O. C. Sampaio R. N. R. (2007). Tratamento da leishmaniose tegumentar Americana *. Bras. Dermatol. 82, 111–124. doi: 10.1590/S0365-05962007000200002

[B12] Martins J. Coelho J. Tadini M. C. de Souza R. O. Figueiredo S. A. Fonseca M. J. V.. (2020). Yangambin and epi-yangambin isomers: New purification method from ocotea fasciculata and first cytotoxic aspects focusing on in vivo safety. Planta Med. 86, 415–424. doi: 10.1055/a-1118-3828 32126582

[B13] MINISTÉRIO DA SAÚDE/SVS/SINAM NET (2021) Leishmaniose tegumentar (LT)—Português (Brasil). Available at: https://www.gov.br/saude/pt-br/assuntos/saude-de-a-a-z/l/leishmaniose-tegumentar/leishmaniose-tegumentar [Accessed May 28, 2022].

[B14] Moro A. Invernizzi N. (2017). A tragédia da talidomida: a luta pelos direitos das vítimas e por melhor regulação de medicamentos. Hist Cienc Saude Manguinhos 24, 603–622. doi: 10.1590/s0104-59702017000300004 29019599

[B15] Neto R. L. M. Barbosa Filho J. M. Sousa L. M. A. Filho F. A. Dias C. S. Rcia M.. (2007). Crude ethanolic extract, lignoid fraction and yangambin from ocotea duckei (Lauraceae) show antileishmanial activity. Z. Naturforsch 62, 348–352. doi: 10.1515/znc-2007-5-605. 17708438

[B16] Neto R. L. M. Sousa L. M. A. Dias C. S. Barbosa Filho J. M. Rcia M. Oliveira R. (2008). Yangambin cytotoxicity: A pharmacologically active lignan obtained from ocotea duckei vattimo (Lauraceae). Z. Naturforsch 63, 681–686. doi: 10.1515/znc-2008-9-1012. 19040107

[B17] Neto R. L. M. Sousa L. M. A. Dias C. S. Filho J. M. B. Oliveira M. R. Figueiredo R. C. B. Q. (2011). Morphological and physiological changes in leishmania promastigotes induced by yangambin, a lignan obtained from ocotea duckei. Exp. Parasitol. 127, 215–221. doi: 10.1016/j.exppara.2010.07.020 20691682

[B18] Penha S. (2010). ESTUDO DA POTENCIALIDADE DA LIGNANA IANGAMBINA E DA QUITOSANA NO TRATAMENTO DA LEISHMANIOSE EXPERIMENTAL EM CAMUNDONGOS SUÍÇOS. Programa Pós-graduação em Prod. Nat. e Sintéticos Bioativos, Univ. Fed. da Paraíba, 119.

[B19] Ribeiro R. Carvalho F. A. S. Barbosa-Filho J. M. Cordeiro R. S. B. Tibiriçá E. V. (1996). Protective effects of yangambin - a naturally occurring platelet-activating factor (PAF) receptor antagonist - on anaphylactic shock in rats. Phytomedicine 3, 249–256. doi: 10.1016/S0944-7113(96)80062-6 23195079

[B20] Rodrigues F. M. D. Coelho Neto G. T. Menezes J. G. P. B. Gama M. E. A. Gonçalves E. G. Silva A. R.. (2014). Expression of Foxp3, TGF-β and IL-10 in American cutaneous leishmaniasis lesions. Arch. Dermatol. Res. 306, 163–171. doi: 10.1007/s00403-013-1396-8 23922083

[B21] Serra M. F. Diaz B. L. Barreto E. Paula A. Pereira B. Lima M. C. R.. (1997). Anti-Allergic Properties of the Natural PAF Antagonist Yangambin. Planta Med. 63, 207–2012. doi: 10.1055/s-2006-957654. 9225600

[B22] Silveira F. T. Lainson R. Corbett C. E. P. (2004). Clinical and immunopathological spectrum of American cutaneous leishmaniasis with special reference to the disease in Amazonian Brazil: a review. Mem Inst Oswaldo Cruz 99, 239–251. /S0074-02762004000300001 15273794 10.1590/s0074-02762004000300001

[B23] Silveira F. T. Lainson R. de Castro Gomes C. M. Laurenti M. D. Corbett C. E. P. (2009). Immunopathogenic competences of leishmania (V.) braziliensis and l. (L.) amazonensis in American cutaneous leishmaniasis. Parasite Immunol. 31, 423–431. doi: 10.1111/j.1365-3024.2009.01116.x 19646206

[B24] Tibirica E. V. Mosquera K. Abreu M. Ribeiro R. Carvalho F. A. Barbosa-Filho J. M.. (1996). Antagonistic effect of yangambin on platelet-activating factor (PAF)-induced cardiovascular collapse. Phytomedicine 2, 235–242. doi: 10.1016/S0944-7113(96)80048-1 23194622

[B25] WHO (2021) Leishmaniasis. Available at: https://www.who.int/news-room/fact-sheets/detail/leishmaniasis (Accessed May 28, 2022).

